# Social Distancing and Artful Pandemic Survival

**DOI:** 10.3201/eid2611.AC2611

**Published:** 2020-11

**Authors:** Terence Chorba

**Affiliations:** Centers for Disease Control and Prevention, Atlanta, Georgia, USA

**Keywords:** coronavirus disease, COVID-19, social distancing and artful pandemic survival, art science connection, emerging infectious diseases, art and medicine, about the cover, A Tale from The Decameron, John Waterhouse, social distancing, bacteria, viruses, plague, smallpox, quarantine, pandemic, respiratory infections, zoonoses

**Figure:**
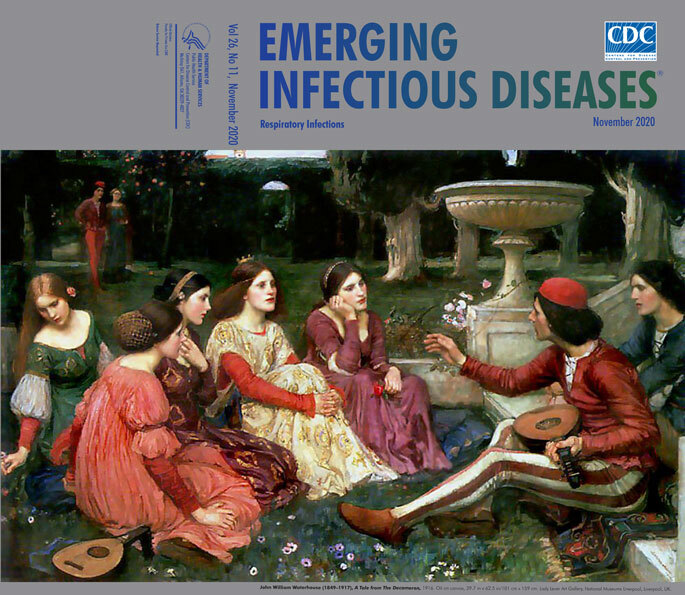
**John William Waterhouse (1849–1917), *A Tale from* *The Decameron*, 1916.** Oil on canvas, 39.7 in x 62.5 in/101 cm x 159 cm. Lady Lever Art Gallery, National Museums Liverpool, Liverpool**,** UK.

“Social distancing” is a relatively novel term, recently popularized in strategies for disrupting transmission of newly identified airborne pathogens, including influenza virus variants, Ebola virus, and coronaviruses. An American cultural anthropologist, Edward T. Hall, Jr., coined the term “social distance” in 1963 to describe a zone of space customarily adopted in many cultures to minimize visual, olfactory, auditory, and tactile stimulation when meeting strangers or mere acquaintances. A PubMed search by term indicates that social distancing as an infectious disease intervention first appeared in a 2005 article about strategies for containing an emerging influenza pandemic. However, awareness of distancing oneself from others as a prevention tool against infection dates to biblical times; reference to removing persons with leprosy from the community was recorded in the Bible (Lev. 13:46, New Jerusalem Version) during the 6th–4th centuries bce. Later, for both plague and smallpox, avoiding contact between diseased and healthy persons became a method for disrupting disease transmission. Beginning in the 14th century, Italian port cities began isolating ships and travelers suspected of carrying plague for periods of 30 days (*trentino*) and later for 40 days (*quarantino*), thus, the English term “quarantine.”

History is replete with examples of artistic genius creatively flourishing in the face of an epidemic. Shakespeare took advantage of London’s intermittent lockdowns in response to plague during 1603–1613 to dedicate time to writing some of his greatest masterpieces; in 1605–1606 alone, when the Globe and other London theaters were closed, he completed *King Lear*, *Macbeth*, *Anthony and Cleopatra, and Timon of Athens*. Earlier, in the mid-1300s, Italian literature was graced with *The Decameron*, a narrative that contains many shorter stories. A humanist writer and poet, Giovanni Boccaccio started the work in 1347 at the beginning of the Black Death, the global epidemic of bubonic plague that peaked in Europe until 1351; he completed the work in 1353. The fictitious tale describes 10 young noble women and men who isolate themselves as a group in a secluded Tuscan villa just outside Florence. This exercise in social distancing is an effort to escape the plague that killed half the Florentine population. Daily, over the course of a fortnight, each group member takes a turn as the storyteller, telling a story that follows a specific theme. In all, *The Decameron* is composed of a hundred novellas, many stories within stories.

**Figure 2:**
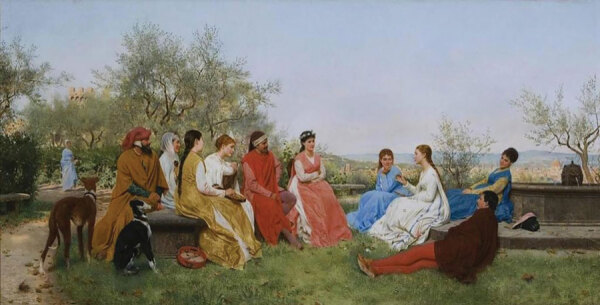
Painting by Raffaello Sorbi (1844–1931), *Decamerone*, 1916. Public domain image. Private collection.

In *A Tale from The Decameron*, the painting on this month’s cover, we see the group in a bucolic setting, devoid of any hint of the devastating plague in nearby Florence. The painting is in the collection of the Lady Lever Art Gallery in Liverpool, known for its Victorian paintings that include works of the Pre-Raphaelite Brotherhood. Pre-Raphaelites were a 19th-century group of English painters who revered medieval culture and the early Renaissance and were prolific in portraying realistic scenes of bygone cultures. *A Tale from The Decameron* was created in 1916 by a realist painter associated with the Pre-Raphaelites, John William Waterhouse (1849–1917). In the painting, Waterhouse depicts five of the young women seated comfortably on cushioned ground, listening to a story being told by one of two young men seated above them on steps. The storyteller, in a red skullcap, is intently speaking, and across his lap lies a mandolin-type instrument that may have some role in the storytelling itself. In the background, two others are strolling together in the villa’s ornate garden. Despite being gathered together, the group members are neither wearing facial masks nor practicing social distancing among themselves. A similar scene from *The Decameron* is portrayed in a painting by Raffaello Sorbi (1844–1931), which features a young woman storyteller (Figure 2); Sorbi, a contemporary of Waterhouse, was a Florentine realist painter, also popular in 19th-century Britain. In both paintings, the opulent surroundings and clothing show the circumstances of a wealthy emerging merchant class in Florence; their resources gave them access to such villas and distance from the urban squalor with its rodents, principally brown rats, which served as a reservoir for fleas that transmitted the epidemic’s pathogen, *Yersinia pestis*.

Whether those living in rural villas rather than in urban settings were any less exposed to the ravages of the Black Death is unknown. However, the perception of the wealthy was that urban flight was a preventive alternative to living in crowded environments where the dead and dying were far more concentrated, where in Boccaccio’s words, people were “*non come uomini ma quasi come bestie morieno*” [dying more like animals than like humans]. Such escape also served a therapeutic psychological purpose, restoring a vision of order, away from the horror and chaos of plague-ridden Florence. In the limited understanding of the 14th century, such escape warded off bad humors that would otherwise increase one’s susceptibility to plague.

In the present pandemic circumstances, we can appreciate that the impacts of communicable diseases on social behavior are somewhat conflicting: increasing altruism and within-group cohesion but decreasing social interactions if there are associated risks for infection. Although much remains for study of combinations of social distancing measures, many observational studies from influenza pandemics have found positive effects of isolating sick persons; quarantining exposed persons; and implementing school and workplace closures, workplace measures to reduce disease transmission, and measures to reduce population density. Early plateauing and abrupt decrease of influenza reports have also been correlated temporally with social distancing efforts in response to SARS-CoV-2 infection.

Fortunately, respiratory spread of *Yersinia* bacteria, principally by coughing or vomiting, occurs only after the disease has progressed. For Boccaccio’s characters, an appreciation of the concept of respiratory transmission of diseases was still a half a millennium away, so the listeners in his masterpiece are portrayed as comfortably sitting closely together without masks. Unfortunately, with the current spread of coronavirus disease via respiratory secretions, we cannot dispense with respiratory protection. However, as with past pandemics, lovers of art, music, and literature hope that necessary measures to avoid infection, including staying at home and engaging in self-isolation and social distancing, will again yield or inspire masterpieces that will enrich our culture and our world.
